# GH1-family 6-P-β-glucosidases from human microbiome lactic acid bacteria

**DOI:** 10.1107/S0907444912049608

**Published:** 2013-02-16

**Authors:** Karolina Michalska, Kemin Tan, Hui Li, Catherine Hatzos-Skintges, Jessica Bearden, Gyorgy Babnigg, Andrzej Joachimiak

**Affiliations:** a Midwest Center for Structural Genomics, Biosciences Division, Argonne National Laboratory, Argonne, Illinois, USA; b Structural Biology Center, Argonne National Laboratory, Argonne, Illinois, USA; c Department of Biochemistry and Molecular Biology, University of Chicago, Chicago, USA

**Keywords:** 6-P-β-glucosidases, glycoside hydrolases, GH1, cellobiose, gentiobiose, salicin

## Abstract

In lactic acid bacteria and other bacteria, carbohydrate uptake is mostly governed by phosphoenolpyruvate-dependent phosphotransferase systems (PTSs). PTS-dependent translocation through the cell membrane is coupled with phosphorylation of the incoming sugar. After translocation through the bacterial membrane, the β-glycosidic bond in 6′-­P-­β-glucoside is cleaved, releasing 6-P-β-glucose and the respective aglycon. This reaction is catalyzed by 6-P-β-glucosidases, which belong to two glycoside hydrolase (GH) families: GH1 and GH4. Here, the high-resolution crystal structures of GH1 6-P-β-glucosidases from *Lactobacillus plantarum* (LpPbg1) and *Streptococcus mutans* (SmBgl) and their complexes with ligands are reported. Both enzymes show hydrolytic activity towards 6′-P-β-glucosides. The LpPbg1 structure has been determined in an apo form as well as in a complex with phosphate and a glucose molecule corresponding to the aglycon molecule. The *S. mutans* homolog contains a sulfate ion in the phosphate-dedicated subcavity. SmBgl was also crystallized in the presence of the reaction product 6-P-β-glucose. For a mutated variant of the *S. mutans* enzyme (E375Q), the structure of a 6′-P-salicin complex has also been determined. The presence of natural ligands enabled the definition of the structural elements that are responsible for substrate recognition during catalysis.

## Abstract 2

The crystal structures of two 6-P-β-glucosidases from the GH1 family were determined in the apo form and in the presence of a 6′-P-salicin substrate, of the reaction product 6-P-β-glucose and of glucose corresponding to the aglycon molecule. The presence of natural ligands enabled the definition of the structural elements responsible for the recognition and hydrolysis of 6′-P-β-glucosides.

## 1. Introduction   

Lactic acid bacteria (LABs) are acidophilic or aciduric Gram-positive bacteria which produce lactic acid as the major end product of carbohydrate metabolism (Kandler, 1983 ▶). Owing to their limited biosynthetic ability, they prefer nutrient-rich environments such as animal oral cavities and intestines as well as other carbohydrate-rich niches. Because their metabo­lism results in food preservation, LABs have been extensively used in industry for the production of fermented products derived from milk, meat, vegetables and other plant materials. Fermentation processes utilizing LABs have two beneficial aspects: bacterial growth lowers both the carbo­hydrate content of the food and its pH. Strong acidification of fermented material inhibits the growth of other food-spoilage microorganisms and potential human pathogens; therefore, fermentation, for example by pickling, enables the prolonged storage of perishable food. In many fermented food products, such as sauerkraut, *Lactobacillus plantarum* is commonly found; it is also used in silage inoculants, where it can rapidly outcompete other bacterial species. The industrial applications of LABs extend beyond food processing, as the lactic acid produced by these microbes constitutes a building block of the eco-friendly polymeric material polylactic acid. Some of the strains that colonize the gastrointestinal tract, such as *L. plantarum* (Ahrné *et al.*, 1998 ▶), have been shown to provide beneficial effects on human and animal health and are marketed as probiotics (Cunningham-Rundles *et al.*, 2000 ▶). The health-promoting role of LABs has been linked to improved digestion, absorption and availability of nutrients. For instance, bacteria that utilize lactose can alleviate the abdominal pain associated with lactose intolerance (Parvez *et al.*, 2006 ▶). LABs are also known to secrete bacteriocins (Nes & Johnsborg, 2004 ▶) and control the level of competing bacterial species, including pathogens, contributing to a ‘healthy’ balance of the gastrointestinal microbial community. However, some LABs also have undesirable features; a few *Enterococcus* and *Streptococcus* strains are pathogenic in humans and animals, such as *S. mutans*, which is the primary causative agent of tooth decay. Other examples of LABs with clinical significance include *L. casei* and *L. rhamnosus*, which have been found to be associated with a variety of infections including endocarditis and bacteremia (Cannon *et al.*, 2005 ▶).

Carbohydrates are the major energy source for LABs and their metabolism utilizes a variety of sugars present in the environment. Carbohydrate uptake mostly occurs through the phosphoenolpyruvate-dependent phosphotransferase system (PEP-PTS; Lorca *et al.*, 2007 ▶). To date, seven phosphotransferase (PTS) families have been identified. Each of them transports different monosaccharides and disaccharides, glycosides, polyols and other sugar derivatives. PTS import is ATP-dependent and comprises energy-coupling proteins and sugar-specific proteins (Saier & Reizer, 1994 ▶). Sugar specificity is provided by a single-chain multidomain protein or a complex of several polypeptides, and sugar translocation is facilitated by a membrane-integrated permease. During membrane crossing, position 6 of the nonreducing end of a carbohydrate molecule is phosphorylated in a phosphate-relay cascade involving several proteins. Subsequently, the phosphorylated sugar is released into the cytoplasm. PTS can transport a number of plant-derived glycosides. In these compounds a sugar is linked to another sugar or to a non­carbohydrate moiety. In β-glucosides a glucose residue is joined to either a second glucose moiety (as in cellobiose and gentiobiose) or to a nonsugar residue (as in salicin, arbutin and esculin). In the cytosol, the imported phosphorylated carbohydrates and their derivatives are further processed by a variety of hydrolases to simpler compounds that can be utilized in diverse cellular processes. Specifically, the β-­glycosidic bond of 6′-P-β-glucoside is cleaved by 6-P-β-glucosidase (6PβGlu; EC 3.2.1.86), releasing 6-P-β-glucose (BG6; glycon), an important cellular precursor, and the remaining portion of the given substrate (aglycon). BG6 then enters the energy-yielding glycolytic pathway and is also utilized in several other key metabolic pathways. The aglycon portion can be further metabolized depending on its chemical nature.

The metabolism of β-glucosides, and of cellobiose in particular, is of significant importance in several branches of the biotechnology industry. It constitutes an intermediate product during the degradation of cellulose, which is the most abundant and renewable carbon source, with potential applications in the development of biofuels and bioplastics. The major bottleneck in cellulose utilization lies in its cost-effective degradation to glucose, which can subsequently be fermented to ethanol, lactic acid or other precursor compounds. The efficient hydrolysis of cellulose requires the concerted action of three different enzymes: endoglucanase (EG), cellobiohydrolase (CBH) and β-glucosidase (βGlu). The first two enzymes are strongly inhibited by cellobiose; therefore, highly active βGlu is one of the critical aspects of cellulose bioconversion. Thus, numerous studies have focused on improving the activity of βGlu in cellulolytic complexes and on cellobiose assimilation in general. It has been documented, for instance, that cellobiose can be directly used in lactic acid fermentation (Abdel-Rahman *et al.*, 2011 ▶). There are two classes of βGlu enzymes: one hydrolyzes β-glucosides, while the other can utilize phosphorylated derivatives of β-­glucosides.

The enzymes that catalyze the cleavage of 6′-P-β-glucosides belong to two families of glycoside hydrolases (GHs): GH1 and the unusual GH4 family that requires NAD^+^ and Mn^2+^ for activity (Varrot *et al.*, 2005 ▶). The GH1 family consists of hydrolases with a number of enzymatic activities and three-dimensional structures have been determined for several representatives of this family, revealing their overall structure and details of the glycon (subsite −1) and aglycon (subsite +1) binding sites, but no such information has been obtained for the 6-P-β-glucosidase subfamily. Here, we report five crystal structures of 6-P-β-glucosidases belonging to the GH1 family from LABs, namely *L. plantarum* WCFS1 (LpPgb1) and *S. mutans* UA159 (SmBgl). For the latter enzyme, we have determined three structures corresponding to complexes with a sulfate ion (1.7 Å resolution), with BG6 (1.5 Å resolution) and with 6′-­P-salicin (PSC; E375Q mutant; 2.5 Å resolution). The two structures of LpPgb1 represent an apo form (apo LpPgb1; 2.3 Å resolution) and a phosphate- and β-glucose-bound form (1.5 Å).

## 2. Materials and methods   

### 2.1. Bioinformatics analysis   

The Conserved Domain Database (Marchler-Bauer *et al.*, 2011 ▶) was used to retrieve GH-family members from the NCBI RefSeq database (March 2012 release; Pruitt *et al.*, 2007 ▶) of complete microbial genomes. A set of 70 position-specific scoring matrices (PSSMs) built from the Glyco_hydro Pfam family was used as a query with an *E*-value of threshold 0.0001. The summary table was generated from the result set using a minimum 90% overall coverage of the PSSM in the query sequences. 6-P-β-glucosidase members of the surveyed LABs were identified with a Protein Cluster profile (PRK09589, CelA) as a query. The resulting ∼300 sequences were filtered by profile coverage and clustered using the *CD-­HIT* program (Li & Godzik, 2006 ▶), with an identity cutoff of 0.9 to reduce sequence redundancy for the phylogenic analysis. A multiple alignment was constructed from the representative set of 126 sequences using the *MUSCLE* program (Edgar, 2004 ▶) with default parameters. The alignment was used for maximum-likelihood tree reconstruction by the *FastTree* program (Price *et al.*, 2010 ▶) with default parameters (JTT evolutionary model, discrete gamma model with 20 rate categories). The same program was used for the calculation of bootstrap values.

### 2.2. Cloning   

The 6PβGlu genes from *L. plantarum* WCFS1 (accession No. YP_004888459.1) and *S. mutans* UA159 (accession No. NP_721937.1) were amplified by PCR using genomic DNA as a template and the following primers: 5′-TACTTCCAATCCAATGCCATGACGATTAAAGGACGAGCGTTTCCA-3′ and 5′-TTATCCACTTCCAATGTTACTACTCAATTTCGGCACCATTTGTCG-3′ for *L. plantarum pgb*1 and 5′-TAC­TTCCAATCCAATGCCATGTCTAAATTACCTGAAAATTTTCTCTGGGG-3′ and 5′-TTATCCACTTCCAATGTTATTAAATGTCATCTCCATTTGAAGCAATGACTTCT-3′ for *S. mutans bgl*. The PCR products were cloned into the pMCSG7 plasmid (Donnelly *et al.*, 2006 ▶) according to the ligation-independent cloning procedure (Aslanidis & de Jong, 1990 ▶; Eschenfeldt *et al.*, 2009 ▶). This vector introduces an N-­terminal His_6_ tag followed by a TEV protease recognition site. The expression vectors pAPC100114 and pAPC100193 bearing the *L. plantarum pgb*1 and *S. mutans bgl* genes, respectively, were transformed into the *E. coli* BL21 (DE3) Magic strain. An E375Q point mutation was introduced into the *S. mutans bgl* gene based on Polymerase Incomplete Primer Extension (PIPE) cloning (Klock & Lesley, 2009 ▶). The efficiency of the creation of cohesive ends was enhanced by T4 polymerase treatment of the amplified plasmid. Briefly, a plasmid carrying the *S. mutans bgl* gene was PCR-amplified by KOD Hot Start polymerase in the presence of 1 *M* betaine and the primers 5′-TTCATTGTTCAGAATGGCTTTGGAGCC­ATTGATCAAG-3′ and 5′-AGCCATTCTGAACAATGA­AAAGCGGTAAATGATACATGTCAG-3′. The unpurified PCR product was digested with T4 polymerase without any dNTPs according to Dieckman *et al.* (2002 ▶). The T4-treated mixture was transformed into the *E. coli* BL21 (DE3) Magic strain. Plasmid purified from a single colony was sequenced at the University of Chicago Cancer Research DNA Sequencing Facility.

### 2.3. Expression and purification   

To produce selenomethionine-labeled LpPgb1 and SmBgl, the bacterial cultures were grown at 310 K and shaken at 200 rev min^−1^ in enriched M9 medium (Donnelly *et al.*, 2006 ▶) until an OD_600_ of 1 was reached. Selenomethionine (SeMet) and a mixture of amino acids inhibiting the metabolic pathway of methionine synthesis were added (Van Duyne *et al.*, 1993 ▶; Walsh *et al.*, 1999 ▶) and the cultures were transferred to 277 K for 1 h. Subsequently, the cultures were transferred to 291 K and protein expression was induced with 0.5 m*M* (LpPgb1) or 1 m*M* (SmBgl) isopropyl β-d-1-thiogalactopyranoside (IPTG). The cells were incubated overnight, harvested and resuspended in lysis buffer [500 m*M* NaCl, 5%(*v*/*v*) glycerol, 50 m*M* HEPES–NaOH pH 8.0, 20 m*M* imidazole, 10 m*M* β-­mercaptoethanol]. The SeMet-labelled proteins were purified as described previously (Kim *et al.*, 2004 ▶). Specifically, the protocol for LpPgb1 purification included immobilized metal-affinity chromatography (IMAC) on an ÄKTAxpress system (IMAC-I; GE Healthcare Life Sciences) followed by His_6_-tag cleavage using recombinant His-tagged TEV protease and a second IMAC step (IMAC-II) to remove the protease, the uncut protein and the affinity tag. The purification of SmBgl also included size-exclusion chromatography performed on a HiLoad 26/60 Superdex 200 column (GE Healthcare Life Sciences) between IMAC-I and IMAC-II. The native (*i.e.* not SeMet-labeled) LpPgb1, SmBgl and E375Q SmBgl proteins were obtained analogously to the SeMet-labeled proteins except for the absence of SeMet from the growth media. The pure proteins were concentrated using Amicon Ultra filters (Millipore, Bedford, Massachusetts, USA) in 20 m*M* HEPES–NaOH pH 8.0, 250 m*M* NaCl, 2 m*M* dithiothreitol (DTT).

### 2.4. Oligomeric state determination using size-exclusion chromatography (SEC)   

Size-exclusion chromatography to determine the oligomeric state of the two proteins was performed on an ÄKTAprime plus workstation using a Superdex 200 16/60 column (GE Healthcare Life Sciences) in a buffer consisting of 20 m*M* HEPES–NaOH pH 8.0, 250 m*M* NaCl, 2 m*M* DTT. The column was equilibrated and calibrated using standard proteins from the HMW Gel Filtration Calibration Kit (GE Healthcare Life Sciences). The following proteins were prepared in running buffer at a concentration of 5 mg ml^−1^ to determine a calibration profile: ferritin (440 kDa), aldolase (155 kDa), conalbumin (75 kDa) and ovalbumin (43 kDa) (GE Healthcare Life Sciences). Elution volumes were noted and a linear regression analysis was applied to the standards. The proteins (∼3 mg ml^−1^ each) were resuspended in the running buffer and analyzed under the same conditions as the standards at a flow rate of 1 ml min^−1^. Analysis of the elution profiles of the proteins suggested that both were dimers (Supplementary Fig. S1^1^).

### 2.5. Synthesis of 6′-P-β-glucosides   

Phosphorylation of salicin, cellobiose and gentiobiose (Sigma–Aldrich, St Louis, Missouri, USA) was performed enzymatically in an ATP-dependent reaction catalyzed by β-­glucoside kinase from *Klebsiella pneumoniae* according to the protocol described by Thompson *et al.* (2002 ▶). The purity of the final products was confirmed by NMR spectroscopy. Preparation of β-glucoside kinase from *K. pneumoniae* was carried out following the procedure for LpPbg1 and SmBgl. The purification only involved the IMAC-I step.

### 2.6. Enzymatic assay   

The activities of the purified enzymes were determined by measuring the increased concentration of NADPH in a 6-P-β-glucosidase/glucose-6-P-dehydrogenase (G6PDH) coupled reaction following the procedure developed by Thompson *et al.* (2002 ▶). The assay was carried out in Corning 96-well UV plates (VWR, Radnor, Pennsylvania, USA). Each reaction was performed in triplicate at 298 K in a volume of 100 µl. The assay buffer consisted of 0.1 *M* HEPES–NaOH pH 8.0, 2 m*M* NADP^+^ and 2 U G6PDH (Sigma–Aldrich, St Louis, Missouri, USA) with a gradual increase in substrate concentration from 10 to 2000 µ*M*. The enzyme (native LpPbg1, SeMet-labeled SmBgl or SeMet-labeled E375Q SmBgl) was added to the reaction buffer to a final concentration of 0.02 µ*M*. In the reaction mixture, the substrates (6′-P-cellobiose, 6′-P-gentiobiose or 6′-P-salicin) were hydrolyzed by the purified 6PβGlu to yield 6-P-β-glucose and the aglycon. The oxidation of 6-P-β-glucose is coupled to the reduction of NADP^+^ by G6PDH; the increased absorbance at 340 nm was measured after 10 min (Powerwave Xs2; BioTek, Winnoski, Vermont, USA) to determine the concentration of NADPH. *K*_m_ and *V*_max_ were calculated using the program *GraphPad Prism* (GraphPad Software, La Jolla, California, USA).

### 2.7. Crystallization   

The proteins were crystallized by the sitting-drop vapor-diffusion technique in 96-well CrystalQuick plates (Greiner Bio-One, Monroe, North Carolina, USA). Crystallization drops consisting of 0.4 µl protein solution and 0.4 µl reservoir solution from the MCSG Crystallization Screens (Microlytic, Woburn, Massachusetts, USA) were prepared using a Mosquito liquid dispenser (TTP LabTech, Cambridge, Massachusetts, USA). The protein concentration was 33 mg ml^−1^ for LpPgb1 and 59 mg ml^−1^ for SmBgl. The mixture was equilibrated against 135 µl reservoir solution. The crystals of LpPgb1 appeared at 297 K in conditions consisting of 0.1 *M* sodium acetate/acetic acid pH 4.5, 0.8 *M* NaH_2_PO_4_/1.2 *M* K_2_HPO_4_. Apo LpPbg1 was crystallized at 289 K from 0.6 *M* NaCl, 0.1 *M* MES–NaOH pH 6.5, 20% PEG 4000. Crystals of SmBgl were obtained at 289 K using a solution composed of 0.2 *M* Li_2_SO_4_, 0.1 *M* Tris–HCl pH 8.5, 40% PEG 400. The crystals of SmBgl–BG6 grew at 289 K from a solution consisting of 0.2 *M* trisodium citrate, 20% PEG 3350 with a protein concentration of 50 mg ml^−1^; 5 m*M* 6′-P-gentiobiose was added to the protein stock solution. E375Q SmBgl–PSC was crystallized using 0.1 *M* HEPES–NaOH pH 7.5, 25% PEG 3350, 5 m*M* 6′-P-salicin at 289 K with a protein concentration of 59 mg ml^−1^.

### 2.8. Data collection   

Prior to flash-cooling in liquid nitrogen, the LpPgb1 crystals were cryoprotected in a solution consisting of mother liquor supplemented with 28% [1.55 *M* (*w*/*v*)] sucrose, while for the apo LpPbg1 crystals 25% glycerol served for cryoprotection. The SmBgl crystals did not require additional cryoprotection. 15% ethylene glycol was used as a cryoprotectant for the SmBgl–BG6 crystals. The E375 QSmBgl–PSC crystals were cryoprotected using 20% glycerol. The X-ray diffraction data sets were collected on beamlines ID-19 and BM-19 (for apo LpPbg1) of the Structural Biology Center at the Advanced Photon Source, Argonne National Laboratory. Single-wavelength anomalous diffraction (SAD) data sets were collected from the SeMet-labeled protein crystals at 100 K near the Se *K* absorption edge. The diffraction images were processed with the *HKL*-3000 suite (Minor *et al.*, 2006 ▶). Intensities were converted to structure-factor amplitudes using the *TRUNCATE* program (French & Wilson, 1978 ▶) from the *CCP*4 package (Winn *et al.*, 2011 ▶). The data-collection and processing statistics are given in Table 1 ▶.

### 2.9. Structure solution and refinement   

The structures of LpPgb1 and SmBgl were solved by the SAD method using selenium peak data and the *HKL*-3000 software pipeline (Minor *et al.*, 2006 ▶). *SHELXD* was used for the heavy-atom search and initial phases were obtained from *SHELXE* (Sheldrick, 2008 ▶). The heavy-atom sites were refined and improved phases were calculated by iterations of *MLPHARE* (Otwinowski, 1991 ▶) and *DM* (Cowtan, 1994 ▶). The initial protein models were built in *ARP*/*wARP* (Langer *et al.*, 2008 ▶). Manual model rebuilding was carried out in *Coot* (Emsley & Cowtan, 2004 ▶) and crystallographic refinement was performed in *PHENIX* (Adams *et al.*, 2010 ▶). The structure of LpPgb1 was refined with anisotropic *B* factors for the protein atoms and a glucose molecule. The refinement protocol for SmBgl included TLS refinement with one group per protein monomer (Winn *et al.*, 2001 ▶). The structures of apo LpPbg1, SmBgl–BG6 and E375QSmBgl–PSC were solved by molecular replacement using either LpPbg1 or SmBgl as a template. Apo LpPbg1 was refined in *REFMAC*5 (Murshudov *et al.*, 2011 ▶) using an amplitude-based twin-refinement protocol and TLS parameters (48 groups). The structures of SmBgl–BG6 and E375QSmBgl–PSC were refined in *PHENIX* (Adams *et al.*, 2010 ▶) with the TLS option (13 groups for SmBgl–BG6 and seven groups for E375QSmBgl–PSC). The refinement statistics are shown in Table 1 ▶.

## 3. Results and discussion   

### 3.1. Distribution of glycoside hydrolases in LABs   

We have analyzed the distribution of glycoside hydrolases in a selected set of 69 LABs with sequenced and annotated genomes. The genomes were scanned against a set of 70 Pfam signatures representing 70 GH families. The abundance of GH members varies widely from organism to organism in LABs. There are more than 40 members in *E. faecalis* V583 and only two members in *S. pyogenes* M1 GAS, both of which are strict human pathogens that are responsible for a wide variety of diseases (Supplementary Table S1). In general, a larger number of GH members are found in lactobacilli (*e.g.* *L. plantarum*, *L. rhamnosus* and *L. lactis*) than in streptococci (*e.g.* *S. pyogenes* and *S. suis*). This could be owing to the specific adaptation to the niche that a given LAB occupies. For example, *L. bulgaricus* and *S. thermophilus*, which are protocooperative species, have adapted to a stable and nutritionally rich environment abundant in simple sugars, eliminating the need for GH members capable of processing more complex carbohydrates. At the other end of the scale are highly flexible and versatile species such as *L. plantarum*, which is typically found in a variety of environments such as the human and animal gut and in dairy, meat, vegetable and plant fermentations. The relative abundance of GH1-family members also follows this overall trend. They are most abundant in the previously mentioned *L. plantarum* and in *S. uberis*, a cattle commensal microbe. The 6-P-β-glucosidases described in this manuscript are in close proximity on the phylogenetic tree (Supplementary Fig. S2). The ten predicted 6-P-β-glucosidases from *L. plantarum* WCFS1 and the three from *S. mutans* UA159 are highly dispersed. Our analysis also revealed that while few GH4-family members were found in the surveyed LABs, all of the 6-P-β-glucosidases belonged to the GH1 family. One additional GH1-family member was identified in both *L. plantarum* WCFS1 and *S. mutans* UA159 with high sequence identity. However, these proteins lack key residues in the L8a loop that contribute to the hydrogen-bond network stabilizing the phosphate moiety of the substrate (see below).

### 3.2. Expression, purification and crystallization   

The LpPbg1 protein is one of 11 proteins from *L. plantarum* WCFS1 annotated as 6-P-β-glucosidases (*pbg1*–*pbg11*) and the first one that has been studied (Kleerebezem *et al.*, 2003 ▶; Supplementary Table S1). We have predicted an additional 6-­P-β-glucosidase in this genome. On the other hand, the *bgl* gene of *S. mutans* UA159 encodes one of four predicted 6-P-β-glucosidases. The SmBgl enzyme has previously been implicated in the metabolism of cellobiose, gentiobiose, salicin and amygdalin (Old *et al.*, 2006 ▶). The other two enzymes, BglA (SmBglA) and AscB (SmAscB), are more specific, hydrolyzing esculin and arbutin, respectively (Cote & Honeyman, 2002 ▶; Old *et al.*, 2006 ▶).

Both SeMet-labeled and native LpPgb1 and SmBgl and E375Q SmBgl were expressed and purified for crystallo­graphic and functional studies utilizing the high-throughput pipeline developed at the Midwest Center for Structural Genomics (Kim *et al.*, 2011 ▶). These two enzymes are ∼55 kDa proteins that share 66% sequence identity. They are composed of 478 (LpPgb1) and 477 (SmBgl) residues, including 13 and 15 methionine residues, respectively. To facilitate efficient purification utilizing Ni^2+^-affinity chromatography, all proteins were appended with an N-terminal His_6_ tag which was subsequently removed by TEV protease, leaving only the SNA– sequence as a non-natural protein extension. The final protein yields were 25 mg (SeMet-labeled LpPgb1), 83 mg (native LpPgb1), 42 mg (SeMet-labeled SmBgl), 48 mg (native SmBgl) and 40 mg (native E375Q SmBgl) per litre of culture.

### 3.3. 6-P-β-Glucosidase activity   

LpPgb1 and SmBgl were assayed for glucosidase activity with three glucosides: 6′-P-salicin, 6′-P-gentiobiose and 6′-P-cellobiose. Both enzymes showed hydrolytic activity towards all of these substrates; however, they differed significantly in their kinetic properties. Overall, SmBgl is at least an order of magnitude more active than LpPgb1 towards the tested substrates. We were able to determine the apparent *K*_m_ and *V*_max_ for SmBgl and 6′-P-salicin (*K*_m_ = 1.02 ± 0.07 m*M*, *V*_max_ = 10.38 ± 0.39 m*M* substrate min^−1^ (µ*M* enzyme)^−1^] and 6′-P-gentiobiose [*K*_m_ = 2.73 ± 0.22 m*M*, *V*_max_ = 9.9 ± 0.6 m*M* substrate min^−1^ (µ*M* enzyme)^−1^] (Supplementary Fig. S3). These values fall at the lower end of the range of characterized 6-P-β-glucosidases. Interestingly, the active-site E375Q SmBgl mutant also showed some residual catalytic activity (0.8% of the wild-type enzyme activity detected for gentiobiose). Moreover, LpPbg1 seems to have a different substrate preference to SmBgl (cellobiose > gentiobiose > salicin *versus* salicin > gentiobiose > cellobiose). Taking into account the relatively weak activity against the tested glucosides and the fact that both bacteria possess multiple enzymes with 6PβGlu function, it is likely that we have not identified the substrates that are preferred by these enzymes.

### 3.4. Structure solution   

The structures of both 6-P-β-glucosidases were solved by the SAD method using SeMet-labeled protein crystals. LpPgb1 crystallized in the hexagonal space group *P*622. The asymmetric unit contained one fully ordered protein chain (residues Met1–Glu478). In addition to the protein sequence derived from the gene sequence, the N-terminal alanine residue (Ala0) from the cloning artifact was visible in the electron-density map and was modeled accordingly. The final model also included 590 water molecules, four phosphate anions, one acetate anion and one glucose molecule.

The crystals of SmBgl belonged to the monoclinic system, space group *P*2_1_. The asymmetric unit was occupied by two protein chains: *A* and *B*. The full-length protein (residues Met1–Ile477) was modeled for both; in chain *A* electron density for the N-terminal Ala0 was also visible. In addition to the polypeptide chains, the model contained 573 water molecules, ten sulfate anions, 12 di(hydroxyethyl)ether molecules (PEG) and seven formate anions.

The apo form of LpPbg1 was solved in the trigonal space group *P*3_1_ with six protein chains in the asymmetric unit, 547 water molecules, one glycerol molecule and six chloride ions. The refined atomic model included residues Ala0–Glu478 of chains *A* and *D*, Thr2–Ala334 and Gln350–Glu478 of chain *B*, Thr2–Asp346 and Gly348–Glu478 of chain *C*, Met1–Leu42, Arg49–Ala344 and Gln350–Glu478 of chain *E* and Met1–Thr44, Pro48–Lys343 and Gln350–Glu478 of chain *F*. The crystals are merohedrally twinned with twin operator *k*, *h*, −*l* and a refined twin fraction of 0.28.

SmBgl–BG6 crystallized in the monoclinic space group *P*2_1_ with two protein molecules in the asymmetric unit. The final model consisted of residues Ala0–Ile477 in both chains as well as 1145 water molecules, five ethylene glycol molecules, two formate ions and two BG6 ligands. The 6-P-β-glucose molecules exist in distorted ^4^ *H*_3_ conformations (the Cremer–Pople parameters are ϕ = 227 and 222°, θ = 60 and 70° and *Q* = 0.58 and 0.64 for molecules *A* and *B*, respectively).

The E375Q SmBgl–PSC crystals belonged to the tetragonal system, with one protein molecule present in the asymmetric unit of the *P*4_1_2_1_2 unit cell. In addition to the polypeptide chain consisting of residues Ala0–Ile477, 34 water molecules, two glycerol molecules and one PSC moiety with its glucosyl group in a distorted ^4^ *H*_3_ conformation (the Cremer–Pople parameters are ϕ = 202°, θ = 48° and *Q* = 0.53) were modeled. The quality of all of the crystallographic models was assessed using the *MolProbity* server (Chen *et al.*, 2010 ▶), revealing appropriate stereochemistry (Table 1 ▶).

### 3.5. Overall structure and comparison with other GH1 proteins   

**Figure 1:**
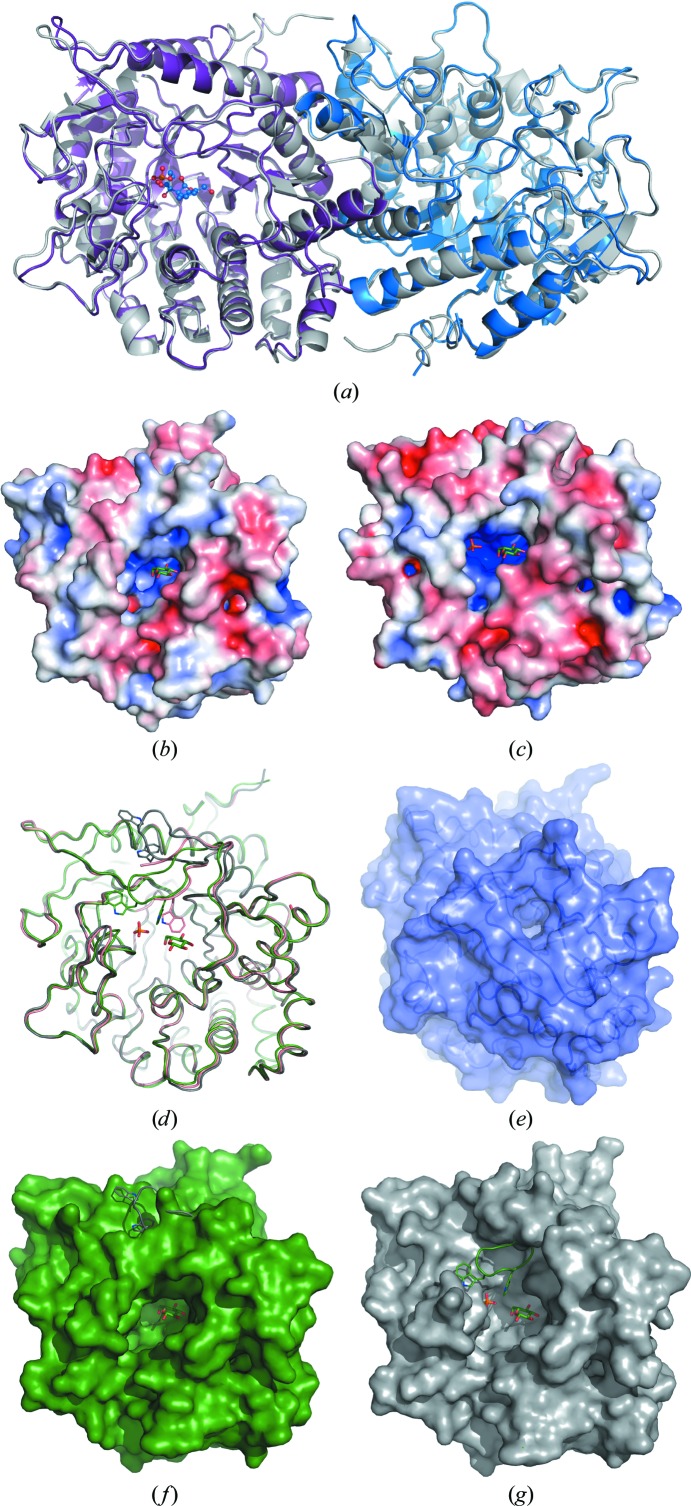
Overall structure of 6-P-β-glucosidase and its comparision with 6-P-β-galactosidase. (*a*) Superposition of the LpPbg1 dimer (grey) and SmBgl (purple and blue). A 6′-P-salicin molecule from the E375Q SmBgl–PSC complex structure is shown in one monomer in ball-and-stick representation. (*b*, *c*) Electrostatic surface potential (calculated using *APBS*; Baker *et al.*, 2001 ▶) of LpPbg1 (*b*) and SmBgl (*c*). The ligands from the LpPbg1 structure are shown for reference. (*d*) Superposition of LpPbg1 (green) with apo LpPbg1 chains *A* (gray) and *C* (pink). Tryptophan residues from the labile loop are shown as line representations. (*e*) 6-P-β-Galactosidase in a surface representation (PDB entry 4pbg). (*f*–*g*) Superposition of LpPbg1 (green) with apo LpPbg1 chain *A* (gray), with either LpPbg1 or apo LpPbg1 shown in a surface representation.

6PβGlu is a single-domain protein that adopts a (β/α)_8_-barrel (TIM-barrel) structure, which is a typical fold of GH1-family members (Fig. 1 ▶). According to the CAZy database (Cantarel *et al.*, 2009 ▶), the GH1 family shows quite diverse enzyme functions and consists of hydrolases with 19 enzymatic activities including 6-P-β-glucosidases (EC 3.2.1.86), β-glucosidases (EC 3.2.1.21), β-galactosidases (EC 3.2.1.23) and 6-P-β-galactosidases (EC 3.2.1.85), amongst others. The tertiary structure has been determined for 31 GH1-family members and is highly conserved. Not surprisingly, a search for structural relatives using *PDBeFold* (also known as *Secondary Structure Matching*; Krissinel & Henrick, 2004 ▶) with LpPgb1 as a template revealed a close similarity to numerous β-glucosidases, with an r.m.s.d. for pairwise C^α^ superpositions of between 1.54 and 1.79 Å and between 29 and 30% sequence identity. A comparable level of similarity has been found between LpPgb1 and other GH1 enzymes such as, for example, dhurrinase from *Sorghum bicolor* (PDB entry 1v02; r.m.s.d. of 1.60 Å for 398 C^α^ atoms; 33% sequence identity; Verdoucq *et al.*, 2004 ▶) and myrosinase from *Brevicoryne brassicae* (PDB entry 1wcg; r.m.s.d. of 1.79 Å for 400 C^α^ atoms; 30% sequence identity; Husebye *et al.*, 2005 ▶). The sequence identity between LpPgb1 and 6-P-β-galactosidase (6PβGal) from *L. lactis*, the only enzyme with 6PβGal activity that has been structurally characterized (PDB entry 1pbg; Wiesmann *et al.*, 1995 ▶), is slightly higher (36%) and structural comparison yields an r.m.s.d. of 1.59 Å for 402 C^α^ atoms. The closest match, with an r.m.s.d. of 0.98 Å for 460 C^α^ atoms and 58% identity, has been found between LpPgb1 and the recently determined structure of 6PβGlu BglA from *Escherichia coli* (PDB entry 2xhy; Totir *et al.*, 2012 ▶).

A comparison between LpPgb1 and SmBgl did not reveal significant structural differences (Fig. 1 ▶). Pairwise superposition of the LpPgb1 C^α^ chain with molecules *A* and *B* of SmBgl gives r.m.s.d.s of 0.66 and 0.60 Å, respectively. The two SmBgl copies superpose with each other with an r.m.s.d. of 0.26 Å. According to the *PISA* predictions (Krissinel & Henrick, 2007 ▶), both proteins form a homodimeric assembly in the crystals, which is in agreement with the size-exclusion chromatographic data in solution (the apparent molecular weights of LpPgb1 and SmBgl are 112 and 114 kDa, respectively, Supplementary Fig. S1).

Superposition of LpPbg1 with apo LpPbg1 yields an r.m.s.d. of between 0.42 and 0.65 Å (for chains *B* and *F*, respectively). Closer inspection of the two structures indicates one major rearrangement that involves movement of loop L6c (Figs. 1 ▶ *d*, 1 ▶ *f* and 1 ▶ *g*), which appears to partially close the active-site cavity in LpPbg1 (Fig. 1 ▶ *d*). In contrast, in the apo LpPbg1 model this loop is shifted towards the solvent, leaving the pocket wide open (Fig. 1 ▶ *g*). The L6c loop bears a conserved Trp349, the side chain of which is a major provider of interactions with the aglycon portion of the substrate (see below). Nevertheless, it seems that the presence of the aglycon moiety is not required for loop closure, since the loop adopts the same closed conformation in SmBgl complexed only with a sulfate ion as in LpPbg1 with phosphate and glucose bound.

In the representatives of the GH1 family the consecutive (β/α) motifs of the conserved (β/α)_8_-barrel core are linked by relatively short loops. Within some of the individual (β/α) repeats additional secondary-structure elements follow the C-­termini of the β-strands. These extensions define unique features for each family subgroup and constitute the active site of the enzyme. In particular, they contribute a set of two key glutamate residues that participate in catalysis (see below; Withers *et al.*, 1990 ▶; Wang *et al.*, 1995 ▶; Moracci *et al.*, 1996 ▶). A long extra C-terminal segment built of two loops and a β-­hairpin provides the elements involved in phosphoryl-moiety binding within its coiled part (L8a loop; see below). Loops L1d and L6b form the entrance to the substrate-binding pocket. Based on the available crystal structures and sequence alignment (Supplementary Fig. S4), the long capping L1d loop seems to be unique to 6-P-β-glucosidases, but even within this subfamily its length and sequence varies. Therefore, it is likely that this region adopts a different conformation in some 6-P-β-glucosidases compared with the LpPgb1 and SmBgl structures. Also, an insertion within the S6–H6 repeat varies among the GH1 enzymes, in particular within its central portion, loop L6b. In 6PβGal there is an additional β-hairpin connected by a long loop that blocks the entrance to the active site. In this closed state the enzyme can only release the aglycon product (glucose), while neither the glycon portion of the product (6-­P-β-galactose) nor the substrate (6-P-β-lactose) can pass through when the gate is closed (Fig. 1 ▶ *e*; Wiesmann *et al.*, 1997 ▶). 6-P-β-glucosidases do not possess this lid motif. As a consequence, the active-site cavity is quite open, with a cross-section of about 20 × 14 Å (the distances between Pro48 and Leu336 and between Glu333 and Gly348, respectively). A possible small lid may be formed by the L1d and L6c loops (see below). The L1d loop partly overlaps with the 6PβGal extra fragment of the S6–H6 insertion.

### 3.6. Active site   

The GH1-family enzymes utilize a double-displacement mechanism of catalysis with retention of configuration at the anomeric C atom of the glycon moiety (Koshland, 1953 ▶; Kempton & Withers, 1992 ▶). Two highly conserved glutamate residues are involved in this process. One of them, Glu180 in LpPgb1 (Glu176 in SmBgl), is part of the T*X*NEP motif located at the end of the β4 strand, while the other, Glu375, is part of the I/VTENG motif situated at the C-terminus of the β7 strand. By analogy to related enzymes, Glu180 is predicted to be a catalytic acid/base which protonates a glycosidic O atom in the first step of the reaction to facilitate the departure of the leaving group (aglycon). At the same time, an electrophilic anomeric C atom is attacked by the nucleophilic Glu375 with the formation of a covalent glycosyl-enzyme intermediate. The second step of the reaction involves Glu180-dependent deprotonation of a water molecule, which subsequently attacks the intermediate, releasing the glycon moiety and the free enzyme. Overall, the active sites of LpPgb1 and SmBgl 6PβGlu are designed to attract negatively charged substrate, with Lys438 contributing to the phosphate binding site, His130 to the glycon binding site and Arg267 to the aglycon binding site (Figs. 1 ▶ *b* and 1 ▶ *c*).

In the 6-P-β-glucosidase structures the key glutamate residues are located at the bottom of a cavity that extends towards the top of the central β-barrel. As mentioned previously, insertions between β-strands and α-helices of the β/α unit constitute the walls of the pocket and provide residues that form the phosphate-binding, glycon-binding and aglycon-binding subsites.

### 3.7. Phosphate binding site   

**Figure 2:**
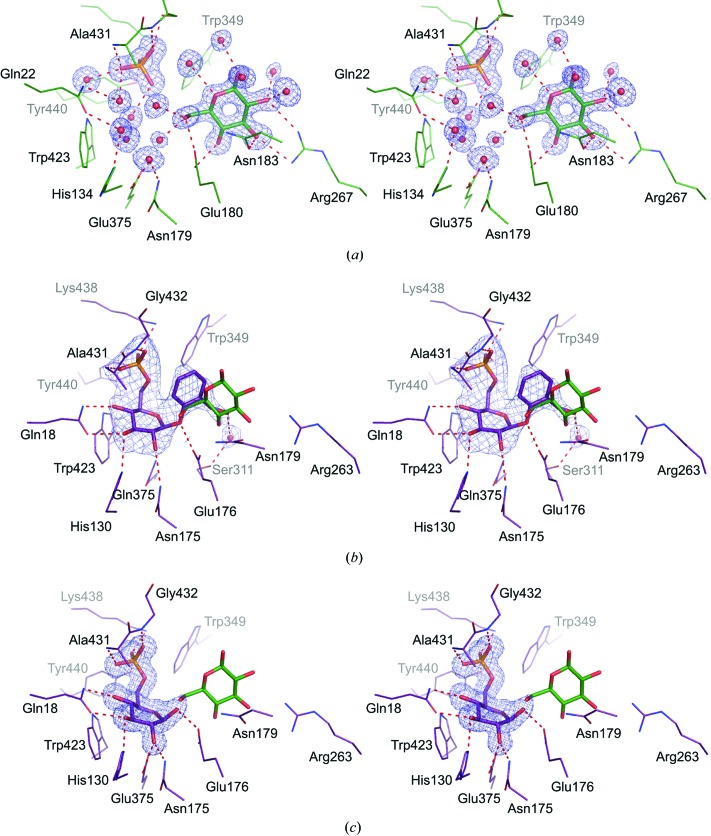
Active site of 6-P-β-glucosidase (stereoview). (*a*) LpPgb1 in complex with a phosphate anion and an aglycon β-glucose moiety. Hydrogen bonds are shown as broken lines. (*b*) E375Q SmBgl in complex with 6′-P-salicin. For comparison, the aglycon glucose molecule from the LpPgb1 structure is shown in green. (*c*) SmBgl in complex with 6-P-β-glucose. All ligands are shown as 2*F*_o_ − *F*_c_ electron-density maps contoured at the 1σ level.

**Figure 3:**
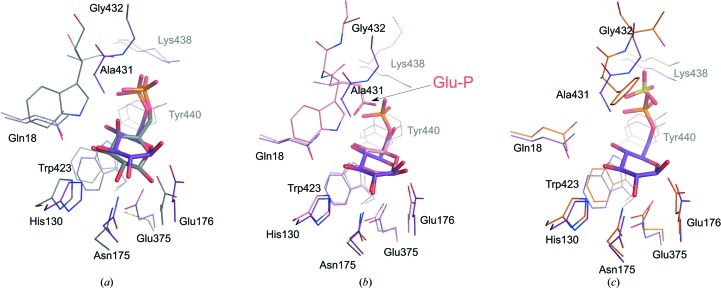
Superposition of the phosphate- and glycon binding sites. The active sites of SmBgl–BG6 (purple) with (*a*) 6-P-β-galactosidase from *L. lactis* in complex with 6-P-β-galactose (gray; PDB entry 4pbg), (*b*) β-glucosidase from an uncultured bacterium in complex with β-glucose (pink; PDB entry 3fj0) and (*c*) 6-P-β-glucosidase A from *E. coli* in complex with a sulfate ion (PDB entry 2xhy) are shown.

The common unit of all of the 6′-P-β-glucoside substrates is BG6. This moiety is recognized by two subsites. One of them is a phosphate binding site, which is unique to 6-­P-β-glucosidases and, to some extent, 6-­P-­β-galacto­sidases. The second is a glucose binding site that is shared with other β-gluco­sidases. The phosphate-binding subsite has been identified in both structures. Substrate-bound and product-bound complexes of SmBgl contain a phosphoryl group attached to the glucose ring occupying the phosphate-dedicated cavity (Figs. 1 ▶, 2 ▶ and 3 ▶). In the LpPgb1 structure this position is occupied by a phosphate anion, while in the sugar-free *Streptoccocus* homolog it is occupied by a sulfate anion. In LpPgb1, the phosphate moiety interacts with the side chains of Lys438, Tyr440 and Ser432 (Figs. 2 ▶ and 3 ▶). Additional anchoring points are provided by the main-chain amides of Ala431 and Ser432. An analogous set of interactions links the anion (or a phosphoryl group) in SmBgl (E375Q SmBgl–PSC), with the exception of Ser432, which is substituted by Gly432, resulting in the elimination of one hydrogen bond. All of these residues belong to loop L8a inserted within the C-­terminal (β/α) motif. This region, which corresponds to the Ala430–Tyr440 fragment in LpPgb1, differs noticeably in length, sequence and spatial arrangement between GH1 members (Supplementary Fig. S4). First of all, the loop is one residue longer in 6-P-β-glucosidases than in β-glucosidases or (6-P)-β-galactosidases, which do not possess an equivalent of the Gln/Glu435 residue. Moreover, 6-P-β-glucosidases and 6-P-β-galactosidases usually contain serine instead of Ala430, while β-glucosidases and β-galactosidases have an invariant phosphomimetic glutamate residue (here called Glu-P). Its side chain occupies the position of the phosphate anion in 6-P-β-glucosidases (Fig. 3 ▶). Therefore, this glutamate plays a key role in discrimination between phosphorylated and nonphosphorylated substrates. In addition, it anchors a glycon moiety of the nonphosphorylated glucosides by hydrogen bonds. Ala431 is conserved amongst 6-P-β-glucosidase family members. Clear exceptions to this rule are BglA from *E. coli*, which contains phenylalanine, and an enzyme from *Fusobacterium mortiferum*, which bears a tryptophan. The sequence of the latter protein generally seems to be more similar to 6-P-β-galactosidases; however, biochemical experiments did not indicate such activity (Thompson *et al.*, 1997 ▶). β-Glucosidases, 6-P-β-galactosidases and β-galactosidases have a conserved tryptophan residue which, considering their function, is part of the glycon binding site rather than the phosphate binding site (see below).

The consequences of the differences in the primary and secondary structures of the L8a loop are threefold. Firstly, the substitution of Ala431 by Trp affects the ability of the enzyme to bind galacto-derived ligands (discussed below). Secondly, the L8a loop determines substrate selectivity with respect to sugar phosphorylation. Thirdly, it contributes directly to phosphate binding, as illustrated by the comparison between 6-P-β-glucosidases and galactosidases (Fig. 3 ▶). The latter enzyme binds a phosphoryl group exclusively using the side chains of residues equivalent to Lys438, Tyr440 and Ser432. The interactions with the main chain observed in LpPgb1 and SmBgl are not present because the entire L8a loop is pushed away from the phosphoryl group. The more complex hydrogen-bond network that stabilizes phosphate binding in 6-P-β-glucosidases is facilitated by the longer differently coiled L8a loop and the absence of the bulky tryptophan residue.

### 3.8. Glycon binding site   

The glycon binding site, also known as the −1 subsite, is formed by residues Gln22, His134, Asn179, Glu180, Glu375, Trp423 and Ala431 (Gln18, His130, Asn175, Glu176, Glu375, Trp423 and Ala431 in SmBgl; Figs. 2 ▶ and 3 ▶). All of these residues are conserved among the GH1-family glucosidases and galactosidases and have been shown to interact with the carbohydrate molecules in a number of crystal structures. Examples include the structures of 6PβGal from *L. lactis* in complex with 6-P-galactonate (PDB entry 4pbg; Wiesmann *et al.*, 1997 ▶), βGlu from the termite *Neotermes koshunensis* in complex with *p*-nitrophenyl-β-glucopyranoside (PDB entry 3ai0; Jeng *et al.*, 2011 ▶), βGlu BglB from *Paenibacillus polymyxa* in complexes with glucose and thiocellobiose (PDB entries 2o9t and 2o9r, respectively; Isorna *et al.*, 2007 ▶) and βGlu from an uncultured bacterium in complex with glucose (PDB entry 3fj0; Nam *et al.*, 2010 ▶). These studies show the tryptophan residue interacting with the glycon moiety using hydrophobic contacts, while the other residues form hydrogen bonds to hydroxyl groups of the glucose molecule. This is further confirmed by the structures of SmBgl–BG6 and E375Q SmBgl–PSC, in which the O1 hydroxyl group/etheric O atom interacts with Glu176 and O2 is hydrogen bonded to Asn175 (and to Glu375 in the BG6 complex). The O3 and O4 hydroxyl groups are both kept in place by Gln18 and, in the case of O3, also by His130. In the LpPgb1 structure the water molecules occupy similar sites mimicking the O2, O3 and O4 hydroxyl groups and their interactions with the protein (Fig. 2 ▶).

### 3.9. Glucose *versus* galactose binding   

Superposition of the 6PβGal–6-P-β-galactose complex with the SmBgl–BG6 complex indicates that 6PβGlu would not be able to easily accommodate the galactose moiety (Fig. 3 ▶ *a*). The two sugars differ in the configuration at the C4 atom, with the O4 hydroxyl group in an axial position in the *galacto* epimer and an equatorial location in the *gluco* epimer. In 6PβGal (but also in *Sulfolobus solfataricus* β-glycosidase; Gloster *et al.*, 2004 ▶), the axial O4 hydroxyl group is within hydrogen-bonding distance of a conserved tryptophan residue that is localized in the phosphate-binding pocket (see above). In contrast, LpPgb1 and SmBgl contain a much more closely located Ala431 which is not only unable to form an analogous interaction but would clash with the *galacto*-configured O4 hydroxyl group. However, it has been shown that the homologous *E. coli* 6-P-β-glucosidases A and B (BglA and BglB) do recognize a *galacto*-derived substrate, although with significantly lower affinity than its O4 epimer (Witt *et al.*, 1993 ▶). The *E. coli* BglB enzyme shares 51% overall sequence identity with LpPgb1 and, in common with most 6-P-β-glucosidases, contains the alanine residue. In the BglA paralog (57% identity to LpPgb1) Ala is substituted by Phe, which results in an even more dramatic reduction of the enzyme activity towards the *galacto* epimer (Wilson & Fox, 1974 ▶): *V*_max_ for the *galacto*-configured substrate is only 0.12% for BglB and 0.0043% for BglA with respect to the *gluco*-configured substrate (100%). As the structure of the *E. coli* homolog closely resembles the structures of the *L. plantarum* and *S. mutans* enzymes (Fig. 3 ▶ *c*), one can speculate that to facilitate binding of the *galacto*-derived substrate some rearrangement of the L8a loop must occur in order to avoid an unfavorable contact between galactose and the alanine (or phenylalanine) side chain and the axial O4 hydroxyl group.

On the other hand, it has been shown by kinetic and structural studies that β-glucosidases bind *gluco*- and *galacto*-configured ligands equally well (Gloster *et al.*, 2004 ▶) despite the presence of the Trp residue in the Ala431 position. The most significant difference in the binding modes of these stereoisomers lies in the interactions between the protein molecule and the O4 hydroxyl group. The epimeric hydroxyl group can bind either to the O^∊1^ atom of Glu-P and to Gln22 (*gluco* epimer) or to the O^∊2^ atom of Glu-P and the tryptophan residue indole N atom (*galacto* epimer). The latter residue forms a weak hydrogen bond to the O3 rather than the O4 of the glucose moiety in the glucose-bound complex. Therefore, the number of key interactions anchoring a substrate molecule in the pocket remains the same, explaining the similar catalytic efficiency. The almost equal specificity is also facilitated by the fact that the Trp residue does not occupy exactly the same position as Ala431 because the conformation of the main chain in this region differs between βGlu and 6PβGlu (Fig. 3 ▶ *b*).

For 6PβGal, mutation of the tryptophan residue shifts the substrate preference towards *gluco*-derived substrates (Schulte & Hengstenberg, 2000 ▶). Gln22 easily accommodates an equatorial O4, while the alanine residue does not provide an anchor for the *galacto*-based compound, indicating that galactose binding strongly depends on the hydrogen bond between Trp N^∊1^ and O4.

### 3.10. Aglycon binding site   

According to biochemical data, GH1-family 6PβGlus are not specific with respect to the aglycon moiety and can accept various aromatic groups or sugars in the +1 subsite (Thompson *et al.*, 1997 ▶). In the high-resolution LpPgb1 structure, a β-glucose molecule is unambiguously in a ^4^ *C*_1_ chair conformation and binds to the +1 subsite (Fig. 2 ▶). The ligand was most likely acquired during cryoprotection with sucrose solution (1.55 *M*) that must also have contained some glucose. Since the phosphate binding site was already occupied by a phosphate ion, soaked glucose could not be accommodated in the −1 subsite because the sugar O6 hydroxyl group would clash with the anion moiety. Therefore, it was bound in the aglycon-dedicated portion of the active site.

The sugar ring is oriented in such a way that its hemiacetal O5 atom points towards the phosphate-binding loop. The molecule interacts directly *via* hydrogen bonds linking O2 and O3 to the guanidinium group of Arg267 and O3 to Asn183. In addition, Glu180 interacts with O4 and O6. The aglycon-binding network is supplemented by several water-mediated contacts and stacking interactions with Trp349. Anchored by numerous interactions, the glucose moiety is very well positioned in the pocket and its electron-density maps are excellent, showing no signs of disorder (Fig. 2 ▶ *a*).

In the E375 QSmBgl–PSC structure the aromatic moiety of PSC occupies the aglycon site. The ring is kept in place by hydrophobic interactions with Trp349 and a water-mediated hydrogen bond to Asn179 (Fig. 2 ▶ *b*). Superposition of E375Q SmBgl–PSC as well as SmBgl–BG6 with LpPgb1 indicates that β-glucose from the latter structure mimics the aglycon portion of 6′-P-gentiobiose, a molecule with two units of glucose joined by a β-(1→6) linkage. The position of the β-­glucose O6 atom nearly corresponds to the glycosidic O atom of PSC and the O1 atom of BG6.

**Figure 4:**
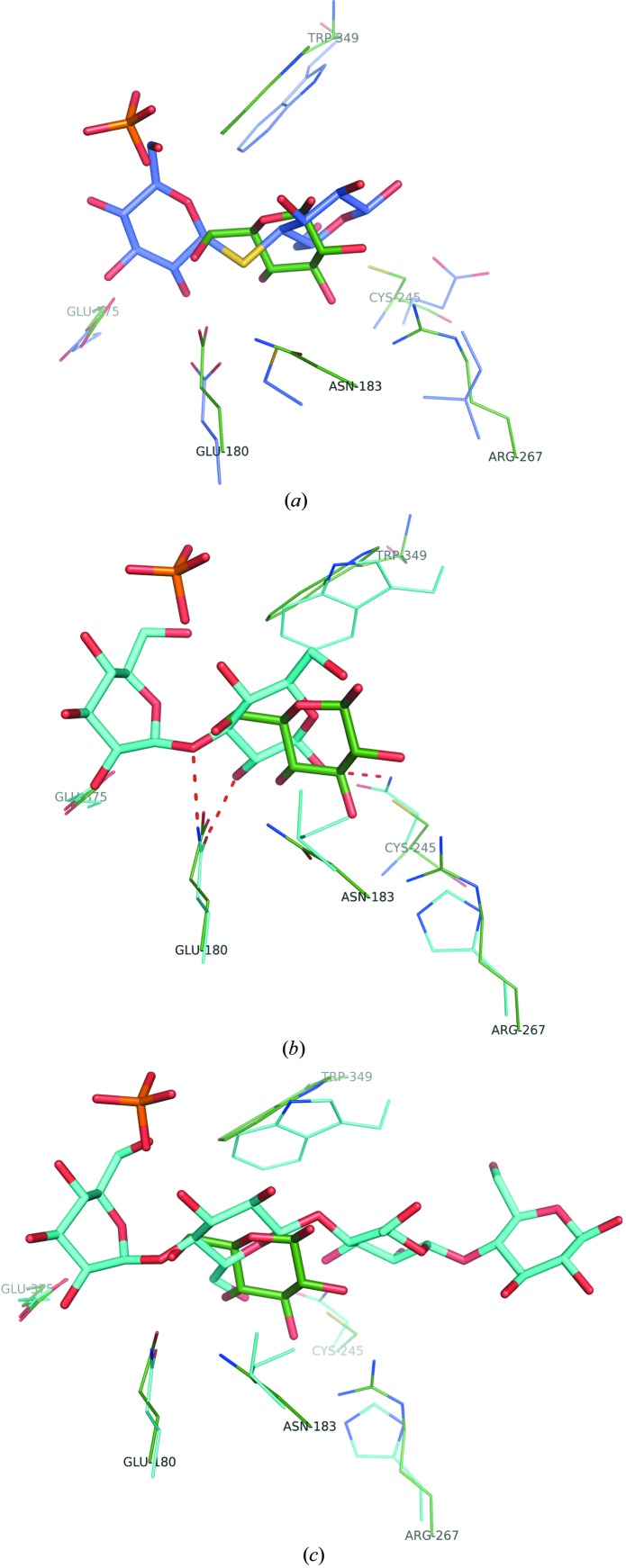
Superposition of the aglycon binding sites. The active sites of LpPgb1 (green) with (*a*) β-glucosidase from *P. polymyxa* in complex with thiocellobiose (blue; PDB entry 2o9r) and β-glucosidase from rice (*b*) in complex with laminaribiose (cyan; PDB entry 3aht; Chuenchor *et al.*, 2011 ▶) and (*c*) in complex with cellotetraose (cyan; PDB entry 3f5j; Chuenchor *et al.*, 2011 ▶) are shown. Hydrogen bonds involving laminaribiose are shown as broken lines.

Previous structural data describing the aglycon binding site (+1 subsite) of GH1 proteins are limited. Most of the available structures of complexes with an aglycon moiety contain an aromatic ring in the +1 subsite (Czjzek *et al.*, 2000 ▶, 2001 ▶; Verdoucq *et al.*, 2004 ▶; Sansenya *et al.*, 2011 ▶). Moreover, in some of them the electron-density maps for the ligands are of limited resolution and do not permit detailed mapping of the protein–ligand interaction (Czjzek *et al.*, 2000 ▶, 2001 ▶). The examples containing +1 sugars are limited to βGlu B from *P. polymyxa* in complexes with thiocellobiose and cello­tetraose (Isorna *et al.*, 2007 ▶) and βGlu from rice in complexes with various oligosaccharides (Chuenchor *et al.*, 2011 ▶). These studies showed that the +1 aglycon moiety is primarily anchored by hydrophobic interactions and water-mediated polar contacts (Chuenchor *et al.*, 2011 ▶; Isorna *et al.*, 2007 ▶). The only exception is laminaribiose [β-(1→3)-linked gluco­disaccharide]; in this case, the aglycon forms two direct hydrogen bonds to the protein molecule (Fig. 4 ▶). In all cases, a conserved Trp residue (Trp349 in the LpPgb1 sequence) serves as a main hydrophobic platform that creates stacking interactions with the +1 sugar ring. The remaining residues shaping the aglycon-binding pocket are not conserved.

Comparison of the LpPgb1–glucose complex with other structures containing the +1 sugar reveals no similarity between the sugar-binding modes. In contrast to other enzymes, LpPgb1 binds its aglycon ligand very tightly through numerous hydrogen bonds. Moreover, the positions and/or orientations of the molecules are different. Generally, the position of the aglycon moiety defines whether the protein–ligand complex represents a Michaelis complex or rather corresponds to a nonproductive substrate/inhibitor-bound state. Orientation of the aglycon moiety, on the other hand, is constrained by the linkage of the glycosidic bond. For example, the structure of βGlu B from *P. polymyxa* in complex with β-(1→4)-thiocellobiose corresponds to a non­productive inhibitor-bound state in which the disaccharide molecule is slightly shifted towards the active-site entrance (as also observed in the complex with cellotetraose). As a consequence, its nonreducing end is localized halfway in-between the −1 and +1 subsites (Fig. 4 ▶). The hemiacetal O5 atom of the reducing-end sugar is oriented in an opposite direction with respect to its LpPgb1-derived glucose equivalent. An analogous orientation of the +1 sugar is observed in the cellotetraose and cellopentaose complexes of βGlu from rice. In these cases, however, the ligands are trapped in the productive positions, with all glucose moieties docked in their respective subsites. Yet another mode of binding is observed with laminaribiose, in which the β-(1→3)-glycosidic linkage enforces a different aglycon orientation. The laminaribiose +1 glucose molecule is rotated 180° about the C3—O5 bond with respect to an analogous molecule from the cellotetraose and cellopentaose complexes. Although the LpPgb1 glucose does not superpose well with any of the above ligands, it has to be noted that the position of its O6 atom nearly corresponds to the glycosidic O atom from laminaribiose (O3) and cello­tetraose/cellopentaose (O4). It has been shown that 6-P-β-glucosidase from *F. mortiferum* is capable of recognizing various β-linkages, with β-(1→6) being among them. As LpPgb1 possesses similar activity, it is likely that the glucose-binding mode in the +1 subsite mimics the binding of the aglycon moiety of 6-P-gentiobiose that contains the β-(1→6)-glycosidic bond.

### 3.11. 6-P-β-Glucosidase isoforms   

The unexpected difference in the enzymatic activities of LpPgb1 and SmBgl is not rationalized by their very similar structures and active-site compositions. This led us to believe that there may be some other factors that are not apparent from the structure but could influence enzyme activity. Both proteins show very high purity and excellent behavior on SDS–PAGE and show monodisperse properties during SEC. However, both proteins show quite extensive heterogeneity on native PAGE gels (Supplementary Fig. S5), suggesting the presence of charge variants (both proteins are exclusively dimers; Supplementary Fig. S1). The LpPgb1 protein appears to be more heterogeneous than SmBgl. The presence of multiple isoforms could be attributed to the deamidation of Asn or Gln residues, a phenomenon that has previously been reported to be associated with spontaneous protein damage or regulation of enzymatic activity for many proteins (Flatmark & Sletten, 1968 ▶; Zomber *et al.*, 2005 ▶; Cox *et al.*, 1999 ▶; Solstad *et al.*, 2003 ▶; Yenpetch *et al.*, 2011 ▶). We have performed mass-spectrometric analysis of the protein bands shown in Supplementary Fig. S5. Indeed, we have observed Asn and Gln deamidation in several tryptic peptides. Although the modifications occur in both proteins, the pattern of deamidation seems to be different. We speculate that that observed for LpPbg1 may be detrimental to its activity, although we are not able to provide a molecular basis for this behavior as the modified residues are mostly localized on the protein surface. Moreover, it is not clear whether the observed heterogeneity is biologically relevant or is an *in vitro* artifact.

## 4. Conclusions   

We have reported several crystal structures of GH1-family 6-­P-β-glucosidases from *L. plantarum* and *S. mutans*. SmBgl structures were determined in complex with a sulfate ion, BG6 and PSC. The structure of LpPgb1 was determined with bound phosphate and β-glucose as well as in the apo form. These structures allow us to define the structural features that are shared with other glucosidases and galactosidases and those that are unique to the 6-P-β-glucosidase subfamily. Both the *L. plantarum* and the *S. mutans* enzymes show hydrolytic activity towards 6′-P-β-glucosides but exhibit surprisingly different kinetic properties and affinities for the substrates. Previous reports have indicated that various LABs show quite different P-β-glucosidase and P-β-galactosidase activities. *L. plantarum* was one of the bacteria that displayed low levels of both 6-P-β-glucosidase and 6-P-β-galactosidase activities in cell suspensions. This is surprising as *L. plantarum* has 11 genes encoding 6-P-β-glucosidases. While their catalytic activities appear to be low, some of them (LpPgb1, LpPbg4 and LpPbg5) show high sequence identity (66–68%) to SmBgl, which appears to have broad substrate specificity. Indeed, our structural studies confirmed a high level of structural homology, including conservation of the active site. The surprisingly low activities of LpPgb1 towards 6′-P-cellobiose, 6′-P-gentiobiose and 6′-P-salicin measured in this study seem to be part of the puzzle. Interestingly, a sequence alignment of all *L. plantarum* proteins annotated as 6-P-β-glucosidases shows that they have an identical −1 subsite (glycon) composition, although their overall pairwise sequence identities are between 31 and 76%. However, their +1 subsites (aglycon) as well as entry to their active sites vary in sequence, including the region between strand β4 and helix α4 of the (β/α)_8_ barrel, which contributes to the +1 subsite and the entrance to the active site. The same structural elements show variability between *S. mutans* 6-P-β-glucosidases. For example, the residues concerned in SmBgl and their corresponding residues in SmBglA are Asn179/Ser183, Phe187/deletion (five residues), Cys241/Met240, Arg263/Asn262, Met314/Met313, Phe316/deletion, Glu332/Asn330 and Gly432/Ser432. Although the pairwise sequence identities between *S. mutans* 6-P-β-glucosidases are 51–54% and their glycon binding-site (−1 subsite) residues are completely conserved, they show different substrate preferences. Considering the conservation of the overall structures and active sites of various 6-P-β-glucosidases, the differences at the +1 subsite and the entrance to the active site are likely to be the determinants of their substrate specificities.

## Supplementary Material

PDB reference: LpPgb1, 3qom

PDB reference: apo LpPbg1, 4gze

PDB reference: SmBgl, 3pn8

PDB reference: SmBgl–BG6, 4f66

PDB reference: E375Q SmBgl–PSC, 4f79

Supporting information file. DOI: 10.1107/S0907444912049608/kw5053sup1.pdf

## Figures and Tables

**Table 1:** Data-collection and refinement statistics Values in parentheses are for the highest resolution shell.

	LpPgb1	Apo LpPgb1	SmBgl	SmBglBG6	E375Q SmBglPSC
Data collection					
Space group	*P*622	*P*3_1_	*P*2_1_	*P*2_1_	*P*4_1_2_1_2
Unit-cell parameters (, )	*a* = 150.6, *c* = 95.9	*a* = 96.1, *c* = 289.1	*a* = 58.9, *b* = 91.0, *c* = 98.6, = 98.7	*a* = 58.7, *b* = 92.4, *c* = 94.4, = 101.3	*a* = 82.3, *c* = 221.2
Temperature (K)	100	100	100	100	100
Radiation source	APS ID-19	APS BM-19	APS ID-19	APS ID-19	APS ID-19
Wavelength ()	0.9792	0.9790	0.9794	0.9792	0.9793
Resolution ()	50.001.50 (1.531.50)	50.002.31 (2.352.31)	36.001.70 (1.731.70)	25.101.48 (1.511.48)	39.002.54 (2.592.54)
Unique reflections	102382	130353	112307	163713	25874
*R*_merge_ †	0.112 (0.650)	0.079 (0.285)	0.115 (0.710)	0.067 (0.589)	0.087 (0.636)
*I*/(*I*)	33.2 (3.7)	21.8 (2.75)	26.8 (1.9)	27.1 (1.9)	25.8 (2.3)
Completeness (%)	99.9 (100)	99.6 (96.7)	98.6 (97.2)	99.7 (96.5)	99.4 (100)
Multiplicity	11.7 (11.4)	4.4 (2.8)	4.1 (4.0)	4.6 (3.1)	6.7 (6.8)
Refinement					
Resolution ()	31.001.50	30.02.31	35.901.69	25.101.48	39.002.54
Reflections (work/test set)	101314/1031	129000/1248	100643/5294	155447/8214	24479/1312
*R*_work_/*R*_free_ ‡	0.118/0.134	0.174/0.213	0.163/0.193	0.162/0.185	0.180/0.229
No. of atoms					
Protein	3993	23032	7846	7925	3902
Ligands	36	12	164	58	36
Water	589	547	565	1145	34
Average *B* factor (^2^)					
Protein	12.7	42.1	37.3	18.5	69.4
Ligands	28.2	52.7	60.4	23.8	79.0
Water	25.9	38.9	39.5	30.8	57.6
R.m.s. deviations from ideal§					
Bond lengths ()	0.015	0.013	0.006	0.006	0.007
Bond angles ()	1.455	1.302	0.939	1.060	1.058
Clashscore [percentile]¶	1.84 [99th]	8.13 [97th]	9.25 [73rd]	5.36 [91st]	13.08 [91st]
Poor rotamers¶ (%)	0.75	1.85	1.99	1.74	4.98
Ramachandran statistics of / angles¶ (%)					
Most favored	99.2	97.5	97.7	98.3	97.5
Outliers	0	0	0	0	0
PDB entry	3qom	4gze	3pn8	4f66	4f79

† *R*_merge_ = 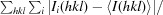 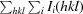, where *I* *_i_*(*hkl*) is the intensity of observation *i* of reflection *hkl.*

‡ *R*_work_ = 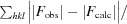 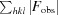 for all reflections, where *F*_obs_ and *F*_calc_ are observed and calculated structure factors, respectively. *R*_free_ is calculated analogously for the test reflections, which were randomly selected and excluded from the refinement.

§ According to Engh and Huber parameters (Engh Huber, 1991 ▶).

¶ According to *MolProbity* (Chen *et al.*, 2010 ▶).

## Footnotes

1 Supplementary material has been deposited in the IUCr electronic archive (Reference: KW5053). Services for accessing this material are described at the back of the journal.
