# Supplementary material for: GH1-family 6-P-β-glucosidases from human microbiome lactic acid bacteria
Source: Acta Crystallogr D Biol Crystallogr. 2013 Feb 16;69(Pt 3):451–63. doi: 10.1107/S0907444912049608 (PMC3605045; doi:10.1107/S0907444912049608)
Supplement: Supplementary file 1 [file d-69-00451-sup1.pdf]

Supplementary Material

Supplementary Table S1. GH distribution.

Species	GH	GH1	GH4
Enterococcus faecalis V583	45	7	1
Lactobacillus plantarum WCFS1	40	11	0
Lactobacillus rhamnosus Lc 705	36	7	3
Lactobacillus plantarum JDM1	35	9	2
Lactobacillus rhamnosus GG	35	8	1
Streptococcus uberis 0140J	34	12	1
Lactococcus lactis subsp. cremoris MG1363	33	9	0
Lactobacillus acidophilus NCFM	32	8	1
Streptococcus pneumoniae 70585	30	6	0
Streptococcus suis BM407	29	5	1
Leuconostoc mesenteroides subsp. mesenteroides ATCC 8293	28	3	0
Lactococcus lactis subsp. cremoris SK11	28	7	0
Streptococcus pneumoniae R6	28	7	0
Streptococcus pneumoniae G54	28	6	0
Lactobacillus johnsonii NCC 533	27	6	1
Lactobacillus casei BL23	26	6	1
Lactobacillus gasseri ATCC 33323	26	7	0
Streptococcus pneumoniae TIGR4	26	6	0
Streptococcus dysgalactiae subsp. equisimilis GGS_124	25	8	0
Leuconostoc citreum KM20	25	6	0
Streptococcus pneumoniae P1031	25	6	0
Streptococcus pneumoniae Taiwan19F-14	25	6	0
Streptococcus gordonii str. Challis substr. CH1	25	5	0
Lactobacillus casei ATCC 334	24	4	2

Streptococcus pneumoniae CGSP14	24	6	0
Streptococcus pneumoniae JJA	22	3	0
Streptococcus mutans UA159	21	4	0
Streptococcus pneumoniae Hungary19A-6	21	4	0
Streptococcus sanguinis SK36	19	4	0
Streptococcus thermophilus LMG 18311	19	6	0
Streptococcus equi subsp. zooepidemicus	18	3	0
Streptococcus equi subsp. equi 4047	17	3	0
Streptococcus equi subsp. zooepidemicus MGCS10565	17	3	0
Streptococcus pneumoniae ATCC 700669	17	3	0
Streptococcus suis 98HAH33	17	3	0
Lactobacillus brevis ATCC 367	17	1	0
Pediococcus pentosaceus ATCC 25745	17	5	0
Streptococcus pyogenes MGAS10270	15	4	0
Oenococcus oeni PSU-1	14	4	0
Streptococcus pyogenes MGAS2096	14	4	0
Streptococcus suis 05ZYH33	13	2	2
Streptococcus pyogenes MGAS10750	13	4	0
Streptococcus pyogenes MGAS315	13	4	0
Streptococcus pyogenes MGAS5005	13	4	0
Streptococcus pyogenes MGAS8232	13	4	0
Streptococcus agalactiae NEM316	12	3	0
Streptococcus pyogenes MGAS10394	11	3	0
Streptococcus pyogenes MGAS6180	11	3	0
Streptococcus pyogenes str. Manfredo	11	3	0
Streptococcus pyogenes NZ131	10	3	0
Lactobacillus sakei subsp. sakei 23K	9	2	0

Lactobacillus helveticus DPC 4571	8	1	0
Streptococcus agalactiae A909	8	1	0
Lactobacillus salivarius UCC118	8	0	0
Streptococcus pneumoniae D39	7	1	0
Lactobacillus fermentum IFO 3956	7	0	0
Lactobacillus reuteri JCM 1112	7	0	0
Streptococcus thermophilus CNRZ1066	6	2	0
Lactobacillus delbrueckii subsp. bulgaricus ATCC BAA-365	6	1	0
Streptococcus suis SC84	5	1	0
Streptococcus agalactiae 2603V/R	4	1	0
Streptococcus pyogenes MGAS9429	4	0	0
Lactobacillus delbrueckii subsp. bulgaricus ATCC 11842	3	0	0
Streptococcus thermophilus LMD-9	3	0	0
Lactobacillus reuteri DSM 20016	2	0	0
Streptococcus pyogenes M1 GAS	2	0	0
Streptococcus pyogenes SSI-1	2	0	0
Streptococcus suis P1/7	2	0	0

Supplementary Figures

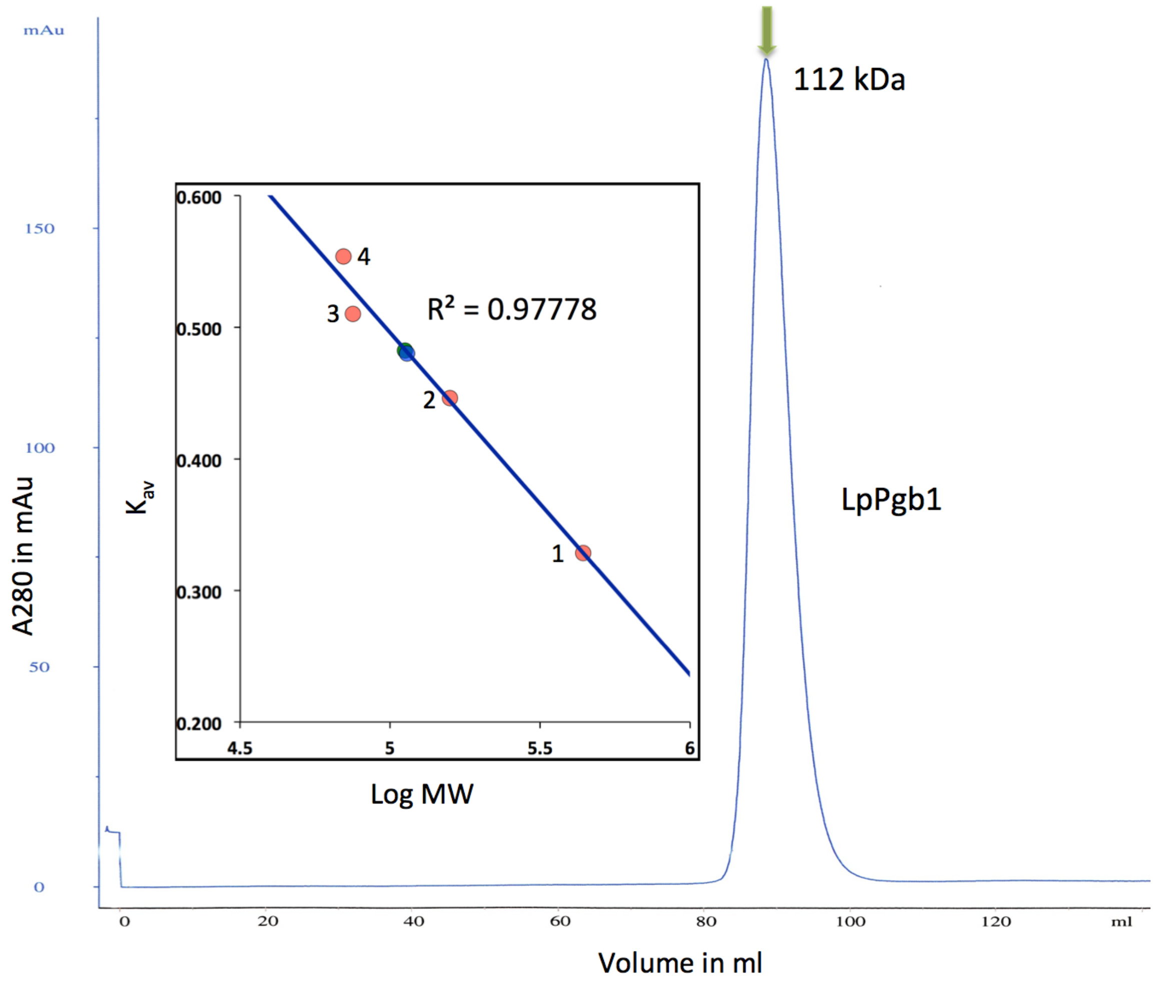

Supplementary Figure S1. Size exclusion chromatography. The absorbance at 280 nm is plotted in absorbance units (AU) versus retention volume in milliliters for LpPgb1. Inserted is a plot of K_{av} coefficient versus logarithm of molecular weight (red circles correspond to standard proteins: (1) ferritin (440 kDa) (2) aldolase (158 kDa), conalbumin (75 kDa), (4) ovalbumin (43 kDa), and the green circle corresponds to LpPgb1 (112 kDa) while the blue circle corresponds to SmBgl (114 kDa)). A single peak corresponding to a dimer is observed for both proteins.

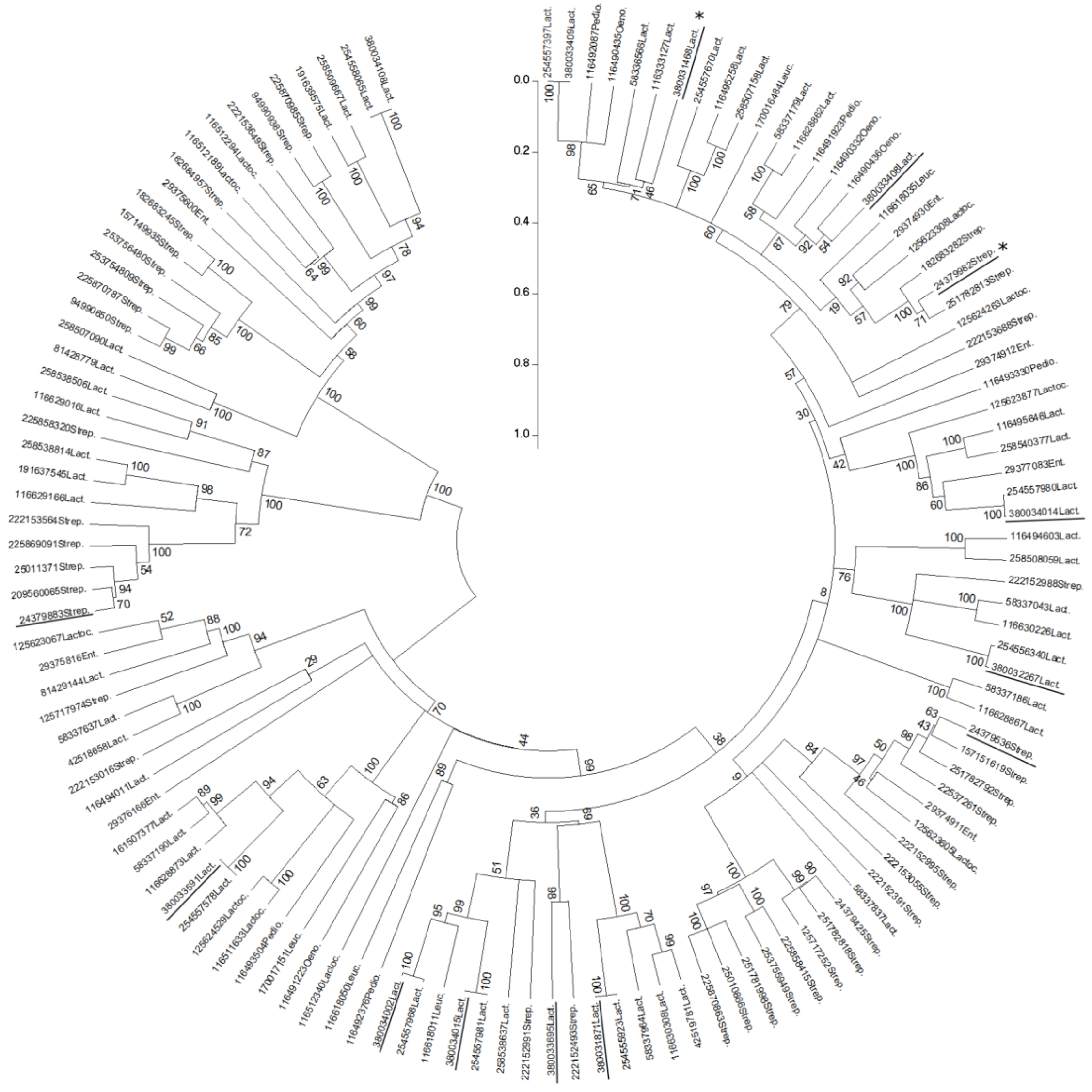

Supplementary Figure S2. Phylogenetic analysis of LAB 6-P-β-glucosidase family members. The tree was constructed using the aligned sequences of 126 CelA family members from 70 LABs with completed genomes. Bootstrap values are indicated. The *L. plantarum* WCFS1 and the *S. mutans* UA159 6-P-β-glucosidase family members are underlined. Each terminal node of the tree is labeled by the Genbank Identifier (GI) number and the name of the genus. The taxonomy codes for the genus are the followings: Lact.: *Lactobacillus*, Strep.: *Streptococcus*, Leuc.: *Leuconostoc*, Pedio.: *Pediococcus*, Ent.: *Enterococcus*, Lactoc.: *Lactococcus*, and Oeno.: *Oenococcus*. The two 6-P-β-glucosidase family members described in this manuscript are labeled with an asterisk.

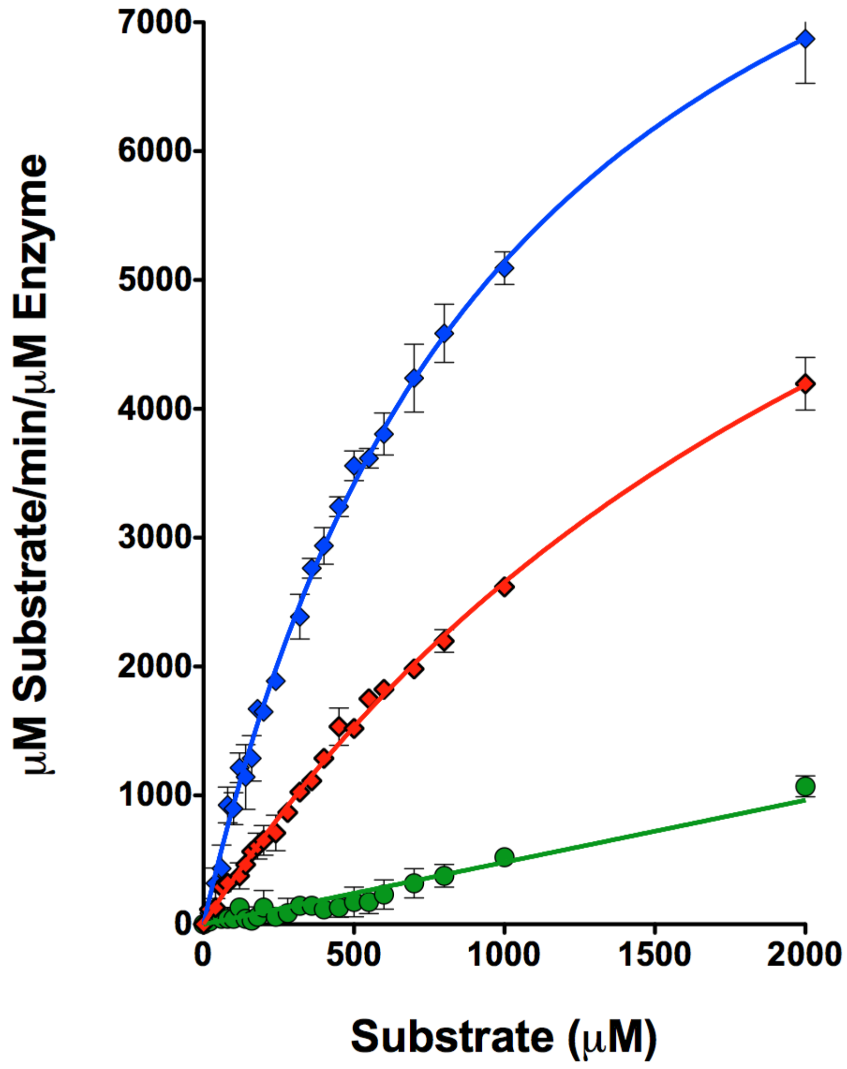

Supplementary Figure S3. Kinetics of 6'-P-β-glucoside hydrolysis. Plot for substrate concentration dependent hydrolysis of 6'-P-salicin (blue), 6'-P-gentiobiose (red) and 6'-P-cellobiose (green) by SmBgl. The graph was generated using the program Prism.

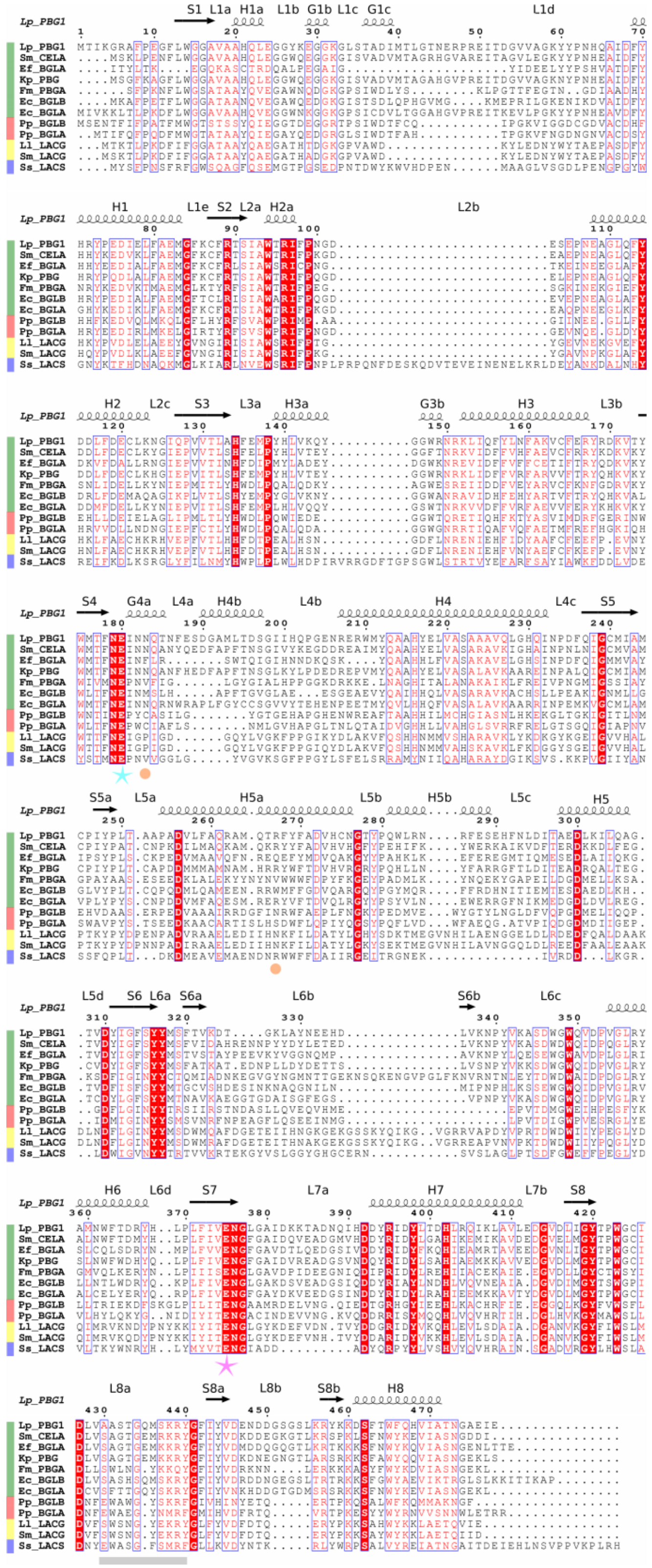

Supplementary Figure S4. Multiple sequence alignment. 6-P-β-glucosidases (green), β-glucosidases (red), 6-P-β-galactosidases (yellow) and a β-galactosidase (blue) were aligned using ClustalW. Catalytic residues (cyan star – acid/base, magenta star – nucleophile) and phosphate-binding loop (grey) are indicated. Residues involved in the glucose binding in LpPbg1 are indicated by an orange circle. Identical residues are highlighted in red, similar

residues are shown as red letters. Secondary structure elements derived from LpPgb1 are depicted. Residues involved in aglycon binding are marked with orange dots. Lp – *L. plantarum*, Sm - *S. mutans*, Ef – *E. faecalis*, Kp – *K. pneumoniae*, Fm - *Fusobacterium mortiferum*, Ec – *E. coli*, Pp – *P. polymyxa*, Ll – *L. lactis*, Ss – *Sulfolobus solfataricus*.

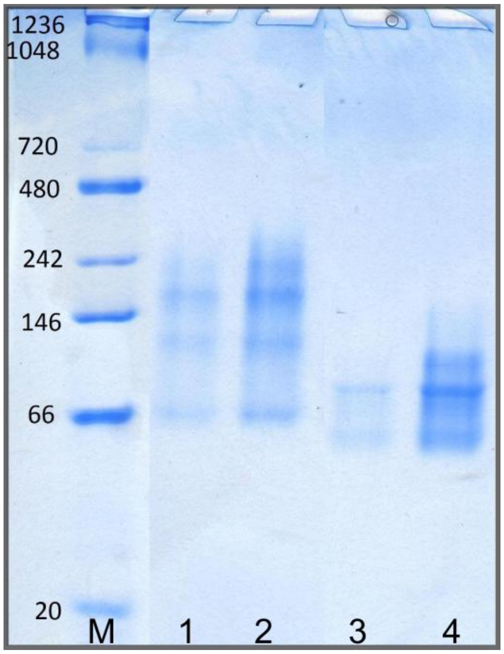

Supplementary Figure S5. Native gel electrophoresis. M – Invitrogen Native Marker (1236 kD – IgM hexamer, 1048 kDa – IgM pentamer, 720 kDa – apoferritin band 1, 480 kDa – apoferritin band 2, 242 kDa – B-phycoerythrin, 146 kDa – lactate dehydrogenase, 66 kDa – bovine serum albumin, 20 kDa – soybean trypsin inhibitor), 1 – LpPbg1 (3.6 μg), 2 – LpPbg1 (5.0 μg), 3 – SmBgl (1.4 μg), 4 – SmBgl (4.7 μg).
